# *GBA*-associated PD: chances and obstacles for targeted treatment strategies

**DOI:** 10.1007/s00702-022-02511-7

**Published:** 2022-05-31

**Authors:** Günter Höglinger, Claudia Schulte, Wolfgang H. Jost, Alexander Storch, Dirk Woitalla, Rejko Krüger, Björn Falkenburger, Kathrin Brockmann

**Affiliations:** 1 Department of Neurology, Hannover Medical School, 30625 Hannover, Germany; 2 German Center for Neurodegenerative Diseases (DZNE), Munich, Germany; 3 Department of Neurodegeneration and Hertie-Institute for Clinical Brain Research, Center of Neurology, University of Tübingen, Hoppe-Seyler-Str. 3, 72076 Tübingen, Germany; 4 German Center for Neurodegenerative Disease (DZNE), Tuebingen, Germany; 5 Parkinson-Klinik Ortenau, Wolfach, Germany; 6 Department of Neurology, Rostock University, Gehlsheimer Str. 20, 18147 Rostock, Germany; 7 German Center for Neurodegenerative Diseases (DZNE) Rostock/Greifswald, Gehlsheimer Str. 20, 18147 Rostock, Germany; 8 Department of Neurology, St. Josef-Hospital, Katholische Kliniken Ruhrhalbinsel, Contilia Gruppe, Essen, Germany; 9 Transversal Translational Medicine, Luxembourg Institute of Health (LIH), Strassen, Luxembourg; 10 Translational Neuroscience, Luxembourg Centre for Systems Biomedicine (LCSB), University of Luxembourg, Esch-sur-Alzette, Luxembourg; 11 Parkinson Research Clinic, Centre Hospitalier de Luxembourg (CHL), Luxembourg, Luxembourg; 12 Department of Neurology, Faculty of Medicine, University Hospital Carl Gustav Carus and Carl Gustav Carus, Technische Universität Dresden, 01307 Dresden, Germany

**Keywords:** PD, GBA, Lysosomal, α-Synuclein

## Abstract

Given the clear role of *GBA* in the pathogenesis of Parkinson’s disease (PD) and its impact on phenotypical characteristics, this review provides an overview of the current knowledge of *GBA*-associated PD with a special focus on clinical trajectories and the underlying pathological mechanisms. Importantly, differences and characteristics based on mutation severity are recognized, and current as well as potential future treatment options are discussed. These findings will inform future strategies for patient stratification and cohort enrichment as well as suitable outcome measures when designing clinical trials.

## Introduction

Over the last decades, research in genetically defined forms of Parkinson’s disease (PD) led to the identification of specific pathways underlying the pathophysiology of the disease. Next to defects in vesicular trafficking, mitochondrial and importantly lysosomal dysfunction represent the most relevant pathways (Jankovic and Tan 2020). Studying these early events provide entry points to develop novel therapeutic targets for stratified patient groups as an important step towards precision neurology. The present article exemplifies such strategies focusing on PD patients with different variants in the *glucocerebrosidase* (*GBA*) gene (PD_GBA_). Also, obstacles of translational research into patient cohorts and study designs for clinical trials are discussed.

## *GBA* and Parkinson

### *GBA* variants are the most important genetic risk factor for PD

Biallelic variants in the *GBA* gene cause Gaucher’s disease (GD), the most common lysosomal storage disorder with tissue accumulation of glucosylceramides due to deficiency of the lysosomal enzyme glucocerebrosidase (GCase). Interestingly, about 25% of GD patients report a first- or second-degree relative to present with PD (Goker-Alpan et al. 2004; Halperin et al. 2006). This important clinical observation was the hint to the fact that heterozygous variants in the *GBA* gene are associated with PD. Subsequently, a large multi-centre study across four continents analysed 5691 PD patients of different ethnic origin compared to 4898 controls and confirmed that with an overall odds ratio (OR) of 5.43, heterozygous variants in the *GBA* gene represent the most important genetic risk factor for PD (Sidransky et al. 2009). This has now been confirmed across different ethnic populations with Caucasian, Asian (Japanese, Chinese, Taiwanese), Hispanic, and African ancestry (den Heijer et al. 2020; Neumann et al. 2009; Lesage et al. 2011; Chen et al. 2014; Mahungu et al. 2020).

To date, more than 100 different variants have been associated with the risk of PD. However, the pathogenicity of different variants varies largely (Table 1). Whereas variants classified as severe variants (e.g. p.L444P) show an odds ratio of 10–15 for developing PD and mild variants (e.g. p.N370S) have an odd ratio of ≤ 5 for PD, some variants that are non-pathogenic for GD have been proven to increase the risk for PD e.g. p.E326K and p.T369M (Iwaki et al. 2019; Zhang et al. 2018; Straniero et al. 2020). These variants show the lowest odds ratios and are thus classified as risk variants. Consequently, *GBA*-subgroup classification for PD patients is often based on variant severity according to established genotype risks reported for PD (PD_GBA_severe_, PD_GBA_mild_, PD_GBA_risk_). Interestingly, we see a huge variability of variant distribution among different ethnicities. About 20% of PD patients with Ashkenazi Jewish ancestry carry a *GBA* variant, with the large majority harbouring the mild p.N370S (> 70%), whereas the severe p.L444P variant is identified in about 5%. Together, the two variants account for about 80% of variants in Ashkenazi Jewish PD patients. In non-Ashkenazi Jewish PD patients, p.L444P is detected in about 30–40% of patients and p.N370S in about 20%, together accounting for 50–60% of variants (Sidransky et al. 2009), indicating that about 40% of variants could be missed if focusing solely on p.N370S and p.L444P. These findings highlight the need for full-gene sequencing and stratification according to variant severity. Moreover, penetrance and disease risk in PD_GBA_ are age-dependent (Anheim et al. 2012; Straniero et al. 2020) and further modified by the composite PD-associated polygenetic risk score (PRS) and single-nucleotide polymorphisms in *SNCA*, *CSTB* and *TMEM175*, the two latter genes encoding proteins associated with lysosomal homeostasis and protein clearance (Blauwendraat et al. 2020b).

**Table 1:** Excerpt of variants in the *GBA* gene detected in PD patients stratified by mutation severity

Variant	Legacy name	Suggested PD severity	References
p.S5N	S(-35)N	VUS	PMID: 26000814
p.R8T	R(-32)T	VUS	PMID: 26296077
p.P12S	P(-28)S	VUS	PMID: 26296077
p.K13R	K(‐27)R	VUS	PMID: 18160183 PMID: 17059888
p.I20V	I(-20)V	VUS	PMID: 26422360
p.L25V	L(-15)V	VUS	PMID: 23225227
c.84dupG		Severe	PMID: 15525722 PMID: 16185900
p.G39R	G(-1)R	VUS	PMID: 27397011
c.115+1G>A	IVS2+1G>A	Severe	PMID: 18434642 PMID: 16185900
p.K46E	K7E	VUS	PMID: 19286695
c.149_150insGTAT		Severe	PMID: 28890071
p.V56F	V17F	VUS	PMID: 29140481
p.C62W	C23W	Mild/severe	PMID: 29140481 PMID: 24434810
p.G74A	G35A	VUS	PMID: 28361101
p.R78H	R39H	VUS	PMID: 20425034
p.Y79C	Y40C	VUS	PMID: 29140481
p.R83C	R44C	VUS	PMID: 20425034
c.307+1G>A	IVS3+1G>A	Mild/severe	PMID: 28830825
c.334_338del		Severe	PMID: 25518742 PMID: 32764102
p.V117A	V78A	Mild/severe	PMID: 28030538 PMID: 18338393
p.G119R	G80R	VUS	PMID: 20947659
p.L144R	L105R	Mild	PMID: 22803570 PMID: 19793665
p.G152A	G113A	Mild/severe	PMID: 20947659 PMID: 18338393
p.I158L	I119L	VUS	PMID: 20947659
p.R159W	R120W	Severe	PMID: 17702778 PMID: 16185900
p.R159Q	R120Q	Severe	PMID: 34779914 PMID: 16185900
p.M162T	M123T	Mild	PMID: 22173904 PMID: 17059888
p.S164N	S125N	Severe	PMID: 20947659 PMID: 12838552
p.R170C	R131C	Severe	PMID: 19286695 PMID: 16185900
p.R170S	R131S	VUS	PMID: 18541817
p.T173P	T134P	Mild/severe	PMID: 26296077 PMID: 16185900
p.D179H	D140H	Mild	PMID: 20425034 PMID: 16185900
p.L183V	L144V	VUS	PMID: 22173904
p.R202*	R163X	Severe	PMID: 20425034 PMID: 16185900
p.R202Q	R163Q	VUS	PMID: 18541817
p.Q205*	R166X	Severe	PMID: 29140481
p.V211L	V172L	VUS	PMID: 23225227
p.S212*	S173X	Severe	PMID: 20947659 PMID: 16185900
c.636_637insTTTC		Severe	PMID: 29140481
p.L213P	L174P	VUS	PMID: 17462935
p.S216T	S177T	VUS	PMID: 23225227
p.W223R	W184R	Severe	PMID: 23225227 PMID: 10679038
p.K225R	K186R	VUS	PMID: 19945510
p.N227S	N188S	Severe	PMID: 19433656 PMID: 12204005
p.N227K	N188K	Severe	PMID: 28890071 PMID: 10649495
p.V230G	V191G	Severe	PMID: 19433656 PMID: 20729108
p.G232W	G193W	Severe	PMID: 19433656 PMID: 27042680
p.G232E	G193E	VUS	PMID: 19286695
p.G234W	G195W	Severe	PMID: 28030538 PMID: 16185900
p.G234E	G195E	Severe	PMID: 27717005 PMID: 15967693
p.S235P	S196P	Severe	PMID: 26296077 PMID: 10649495
p.L236F	L197F	Severe	PMID: 21856586 PMID: 16185900
p.K237T	K198T	VUS	PMID: 14728994
p.P240H	P201H	Severe	PMID: 22387070 PMID: 20729108
p.G241R	G202R	Severe	PMID: 20947659 PMID: 16185900
p.Y244C	Y205C	Severe	PMID: 27294386 PMID: 11933202
p.F252I	F213I	Severe	PMID: 19433656 PMID: 16185900
p.F252V	F216V	VUS	PMID: 28030538
p.F255Y	F216Y	Mild	PMID: 20425034 PMID: 16185900
p.L256P	L217P	VUS	PMID: 23225227
p.Y283*	Y244X	Severe	PMID: 29140481
p.F285L	F246L	VUS	PMID: 22282650
p.H294Q	H255Q	Severe	PMID: 19383421 PMID: 16185900
p.R296Q	R257Q	Severe	PMID: 19286695 PMID: 16185900
p.I299T	I260T	Severe	PMID: 22173904 PMID: 15967693
p.R301C	R262C	VUS	PMID: 28030538
p.R301H	R262H	VUS	PMID: 18987351
p.L303I	L264I	Mild/severe	PMID: 25518742 PMID: 29625627
p.G304S	G265S	VUS	PMID: 28030538
c.914delC		Severe	PMID: 26296077 PMID: 16185900
p.P305L	P266L	Severe	PMID: 27717005 PMID: 11783951
p.S310G	S271G	Mild	PMID: 18541817 PMID: 21779299
p.R316C	R277C	Mild	PMID: 22387070 PMID: 22375149
c.953delT		Severe	PMID: 22968580 PMID: 16185900
p.T336S	T297S	VUS	PMID: 27094865
p.Y343C	Y304C	Severe	PMID: 20947659 PMID: 16185900
p.W351R	W312R	Severe	PMID: 28030538 PMID: 22429443
p.L353V	L314V	VUS	PMID: 25518742
p.F355I	F316I	VUS	PMID: 26296077
p.T362I	T323I	Mild	PMID: 20425034 PMID: 1301953
p.L363P	L324P	Mild/severe	PMID: 23588557 PMID: 16185900
p.E365K	E326K	Risk	PMID: 14728994 PMID: 27648471
p.R368C	R329C	Mild	PMID: 14728994 PMID: 17059888
p.R368H	R329H	VUS	PMID: 19383421
p.L375P	L336P	Mild/severe	PMID: 20425034 PMID: 16185900
p.S378L	S339L	VUS	PMID: 21856586
p.G383S	G344S	VUS	PMID: 20425034
p.F386L	F347L	VUS	PMID: 22387070
p.L393P	L354P	VUS	PMID: 23225227
p.W396R	W357R	VUS	PMID: 28830825
p.R398*	R359X	Severe	PMID: 21779299 PMID: 16185900
p.S403N	S364N	Mild/severe	PMID: 20947659 PMID: 11259172
p.I407T	I368T	VUS	PMID: 28361101
p.T408M	T369M	Risk	PMID: 14728994 PMID: 27648471
p.T408=	T369T	VUS	PMID: 28399184
p.N409S	N370S	Mild	PMID: 14728994 PMID: 16185900
p.N409K	N370K	Mild/severe	PMID: 20425034 PMID: 16185900
p.L410I	L371I	VUS	PMID: 20425034
p.V414L	V375L	Mild	PMID: 25518742 PMID: 16185900
p.V414G	V375G	Mild/severe	PMID: 23225227 Farah P Daniel P El Khoury G El Rachkidi RTohme A. Early onset, but late diagnosis of a rare disease. Intern Med Open J. 2019; 3(1): 1–3
p.G416S	G377S	Severe	PMID: 20947659 PMID: 22429443
p.G416D	G377D	VUS	PMID: 28830825
p.W417G	W378G	Severe	PMID: 21856586 PMID: 32764102
p.D419N	D380N	Severe	PMID: 20425034 PMID: 21982627
p.D419A	D380A	Severe	PMID: 19286695 PMID: 16185900
p.D419V	D380V	VUS	PMID: 22812582
c.1263_1317del	RecΔ5	Severe	PMID: 19286695 PMID: 16185900
p.N425K	N386K	Severe	PMID: 24997549 PMID: 33176831
p.P426L	P387L	Mild/severe	PMID: 28361101 PMID: 8937765
p.E427K	E388K	VUS	PMID: 20947659 PMID: 22820396
p.P430L	P391L	Mild/severe	PMID: 25957717 PMID: 16185900
p.N431S	N392S	VUS	PMID: 22812582
p.W432R	W393R	Mild	PMID: 22173904 PMID: 18847161
p.W432*	W393X	Severe	PMID: 24126159
p.V433L	V394L	Severe	PMID: 18434642 PMID: 16185900
p.N435T	N396T	Mild	PMID: 18160183 PMID: 16185900
p.V437I	V398I	Mild	PMID: 22968580 PMID: 17059888
c.1309delG		Severe	PMID: 24997549
p.D448H	D409H	Severe	PMID: 17462935 PMID: 16185900
p.F465V	F426V	VUS	PMID: 28030538
p.P467S	P428S	VUS	PMID: 24997549
c.1439_1445del		Severe	PMID: 22968580 PMID: 22429443
p.K480N	K441N	VUS	PMID: 28361101
p.D482N	D443N	VUS	PMID: 19286695
c.1447-1466delinsTG		Severe	PMID: 24126159 PMID: 16185900
p.L483P	L444P	Severe	PMID: 14728994 PMID: 16185900
p.L483R	L444R	Severe	PMID: 27717005 PMID: 16185900
p.A485T	A446T	VUS	PMID: 28030538
p.A485A	A446A	VUS	PMID: 20947659
p.V486E	V447E	Mild/severe	PMID: 28834018 PMID: 22344629
p.L488L	L449L	VUS	PMID: 28030538
p.P491L	P452L	VUS	PMID: 20947659
p.D492N	D453N	VUS	PMID: 28030538
p.G493D	G454D	VUS	PMID: 30363439
p.V496A	V457A	VUS	PMID: 28830825
p.V496D	V457D	VUS	PMID: 28030538
p.V499L	V460L	VUS	PMID: 26296077
p.V499M	V460M	Mild/severe	PMID: 20425034 PMID: 16185900
p.N501K	N462K	Severe	PMID: 23413260 PMID: 16185900
p.R502C	R463C	Severe	PMID: 19286695 PMID: 16185900
p.R502P	R463P	Mild/severe	PMID: 27717005 PMID: 16185900
p.R502H	R463H	Severe	PMID: 20947659 PMID: 22429443
c.1505+1G>T	IVS10+1G>T	Mild/severe	PMID: 25249066 PMID: 23430543
c.1506-1G>A	IVS10-1G>A	Severe	PMID: 21745757 PMID: 7694727
p.S504P	S465P	VUS	PMID: 23225227
p.K505K	K466K	VUS	PMID: 22387070
p.T521K	T482K	Mild/severe	PMID: 20425034 PMID: 32547927
p.S527T	S488T	VUS	PMID: 22173904
p.I528V	I489V	VUS	PMID: 24126159
p.H529R	H490R	VUS	PMID: 27397011
p.R535C	R496C	Mild	PMID: 19433656 PMID: 16185900
p.R535H	R496H	Mild	PMID: 15525722 PMID: 16185900
p.Q536R	Q497R	VUS	PMID: 17462935
[p.L483P;p.A495P]	RecA456P (L444P + A456P)	Severe	PMID: 19286695 PMID: 9279145
[p.L483P;p.A495P;p.V499=]	RecNciI (L444P + A456P + V460V)	Severe	PMID: 16261622 PMID: 16185900
[p.D448H;p.L483P;p.A495P;p.V499=]	RecTL (D409H + L444P + A456P + V460V)	Severe	PMID: 18434642 PMID: 16185900

Variant position based on NM_001005742. Suggested PD severity mainly based on reported GD severity. Additionally, frameshift and nonsense variants were categorized as "severe". Variants described as pathogenic in GD, but with unknown GD severity were categorized as "mild/severe". Variants described as not pathogenic in GD, but have been detected as risk factors for PD were categorized as "risk". Missense and splice site variants not described in GD and of unknown significance for PD were categorized as "VUS" = variants of unknown significance

### PD_GBA_: severe clinical trajectories with early cognitive decline

Detailed investigation of the phenotypical spectrum, longitudinal trajectories, and rate of progression of motor and non-motor symptoms is of utmost importance to estimate effect sizes and design clinical trials for disease-modifying therapies (duration, sample sizes, progression rates, expected spectrum of symptoms, etc.).

In general, PD_GBA_ show an earlier age at onset compared to PD patients without *GBA* variants (PD_GBA_wildtype_) with a median onset in the early 50s (Sidransky et al. 2009; Blauwendraat et al. 2019). Of note, this effect is not only attributable to *GBA* variants per se, but is driven by *GBA* variant severity and variant burden with severe variants as well as homozygous and compound heterozygous variants predisposing to the youngest age at onset (Malek et al. 2018; Thaler et al. 2017). Moreover, age at onset is further reduced in PD_GBA_ by non-coding variants in *SNCA* and *TMEM175* (Blauwendraat et al. 2020b). Although younger, PD_GBA_ present with a higher prevalence of cognitive impairment and more frequently suffer from additional non-motor symptoms including neuropsychiatric disturbances (depression, anxiety, and hallucination), autonomic dysfunction and sleep disturbances such as REM-sleep-behaviour disorder (RBD) when compared to PD_GBA_wildtype_, (Brockmann et al. 2011; Barrett et al. 2014). These findings have been replicated consistently over the following years in other PD cohorts worldwide, the latest large clinical genome-wide association study in 4093 PD patients (Iwaki et al. 2019). Importantly, *GBA* variants that are classified as severe (PD_GBA_severe_) have been associated with a more aggressive clinical phenotype suggesting a relevant effect depending on *GBA* variant severity (Cilia et al. 2016; Thaler et al. 2018; Petrucci et al. 2020; Lerche et al. 2021a).

Data from longitudinally investigated cohorts of PD_GBA_ confirm findings from cross-sectional evaluations and revealed that PD_GBA_, although younger in age and age at onset, present with an accelerated disease progression in terms of motor impairment and cognitive decline as compared to PD_GBA_wildtype_. Moreover, survival rates are shorter when compared to PD_GBA_wildtype_ (Brockmann et al. 2015b; Cilia et al. 2016). In a British cohort, after 10 years of disease duration, 46% of PD_GBA_ remained dementia-free in comparison to 68% of PD_GBA_wildtype_. After 15 years, 64% of the surviving PD_GBA_wildtype_ remained dementia-free. At that time point, all PD_GBA_ had developed dementia or already died. Mean time to dementia was 8.3 years in PD_GBA_ compared to 13.7 years in PD_GBA_wildtype_. Similarly, at 5 year disease duration, 67.5% of PD_GBA_ had reached HY stadium 3, compared to 43% of PD_GBA_wildtype_. Mean time to Hoehn and Yahr staging 3 was 4.7 years in PD_GBA_ compared to 6.8 years in PD_GBA_wildtype_ (Stoker et al. 2020). Similar results were reported in a large longitudinal cohort of Italian patients with a clearly more aggressive pattern depending on *GBA* variant severity (Cilia et al. 2016). Interestingly, a recent study reports that PD_GBA_ who are treated with deep brain stimulation (DBS) in the subthalamic nucleus (STN) showed an even more rapid cognitive decline compared to PD_GBA_ without DBS as well as PD_GBA_wildtype_ with and without DBS. This finding suggests that the additive effect of *GBA* variants and STN-DBS negatively impact cognition and that presurgical genetic screening should be considered (Pal et al. 2022). Further studies are needed for replication and to evaluate the underlying pathophysiological mechanisms.

The typical motor manifestation of PD is preceded by a prodromal phase that is characterized by a variety non-motor and early motor signs (Berg et al. 2015). Non-motor symptoms include among others hyposmia, autonomic dysfunction, and neuropsychiatric symptoms, whereas reduced arm swing and bradykinesia indicate early motor signs. However, type, prevalence, time of occurrence, and rate of progression of these prodromal symptoms are variable between patients. Given the findings from the manifest disease phase in PD_GBA_ with the pronounced non-motor profile and more rapid disease progression, we retrospectively assessed patient’s perception of their individual prodromal phase before PD diagnosis. Comparing PD_GBA_ and PD_GBA_wildtype_, we could show that: (i) PD_GBA_ demonstrate a higher prevalence of prodromal symptoms and a shorter prodromal phase with almost parallel beginning of non-motor and early motor signs before PD diagnosis. Contrary, PD_GBA_wildtype_ show a long prodromal interval starting with non-motor symptoms long before early motor signs manifested. (ii) PD_GBA_ with severe variants reported the highest total amount of prodromal signs. These findings suggest that complexity of symptoms known from the manifest disease might be present already in the prodromal phase (Zimmermann et al. 2018). Similarly, prospective studies found that prodromal *GBA* variant carriers present with more pronounced deterioration of motor and non-motor symptoms, specifically cognitive decline and hyposmia when compared to healthy controls without *GBA* variant (Avenali et al. 2019; Beavan et al. 2015; Mullin et al. 2019). Another study in patients with REM-sleep behaviour disorder (RBD) reports that *GBA* variants are associated with accelerated phenoconversion to PD and/or dementia in this specific cohort (Honeycutt et al. 2019).

### *GBA* variants are an important genetic risk factor for Dementia with Lewy Bodies (DLB)

**Fig. 1:**
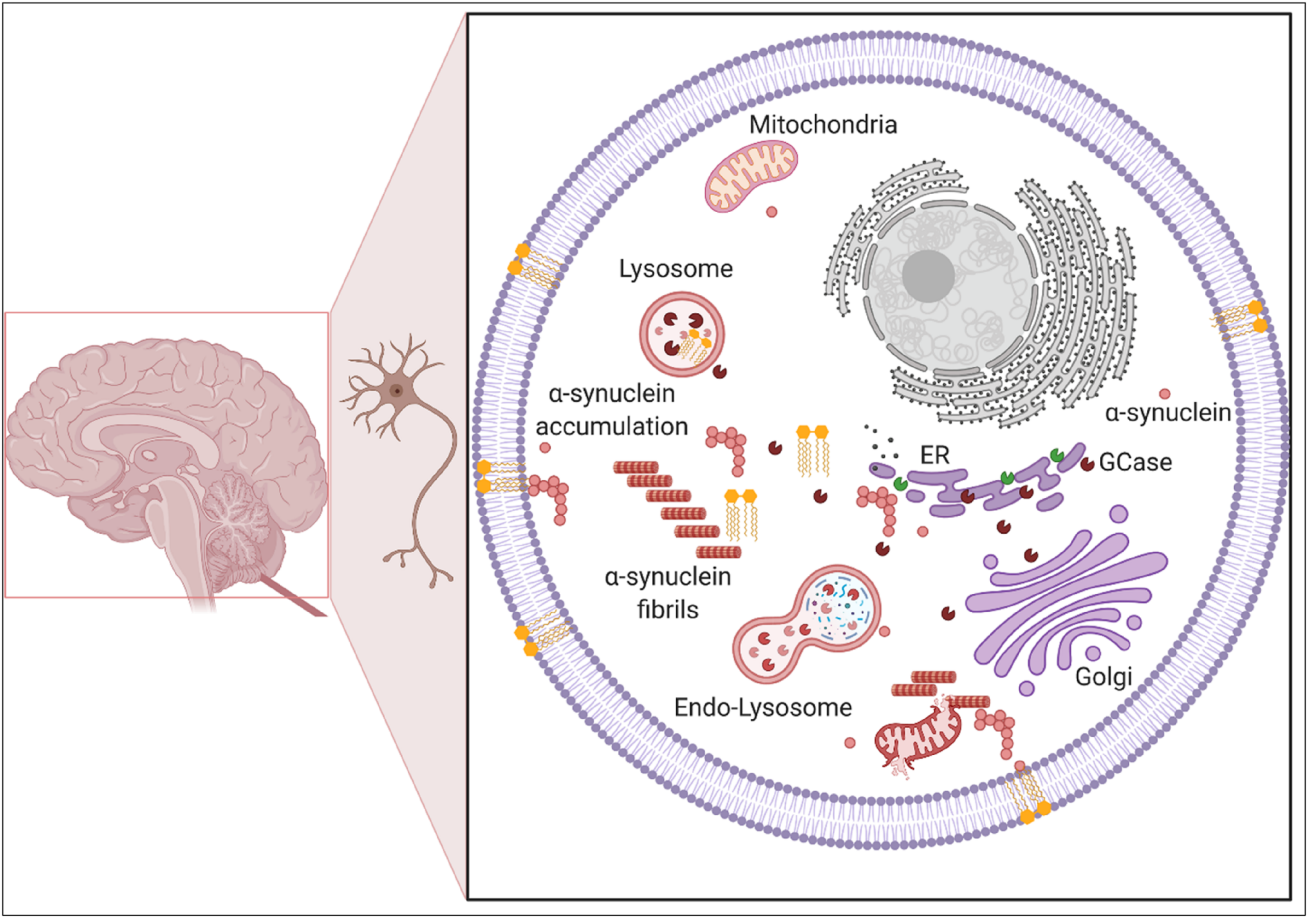
Pathogenic mechanisms underlying PD_GBA_. Loss of lysosomal GCase activity results in impaired autophagy affecting the degradation of both physiological (red dot) and misfolded α-synuclein (red dot complex) resulting in the aggregation of α-synuclein (red strains). *GBA* variants also cause the GCase protein to misfold in the ER (brown enzyme) with impaired trafficking to the lysosome which also affects α-synuclein degradation. Accumulation of GCase substrates (GlcCer and GlcSph, yellow) also causes α-synuclein misfolding and aggregation, as may changes in the lipid homeostasis (both sphingolipids and phospholipids) of cellular membranes (yellow) due to decreased lysosomal function. In PD_GBA_wildtype_, the trafficking of wild-type GCase (green enzyme) can be inhibited by increased levels of α-synuclein (red dot complex) and α-synuclein fibrils (red strains), and contribute GCase deficiency (brown enzyme) irrespective of a *GBA* mutation. This figure was adapted from “Brainstem with Callout” and “Structural Overview of an Animal Cell”, by BioRender.com (2022). Retrieved from https://app.biorender.com/biorender-templates

The important finding that PD_GBA_ shows pronounced and early development of dementia prompted the community to perform a large multicenter analysis across 11 centres evaluating *GBA* variants in 721 cases with DLB, which represents a clinico-pathological continuum to PD. With an even higher OR than seen in PD, *GBA* variants are also strongly associated with DLB (8.28). Similar to PD, *GBA* variants predispose to an earlier age at onset, more pronounced disease severity/progression and rather “pure” form of DLB without concomitant Alzheimer’s profile as defined by CSF p-tau/Aβ1-42 ratio (Nalls et al. 2013; van der Lee et al. 2021). This study further supports *GBA* variants as a significant genetic risk factor for synucleinopathies and confirmed the overall impression that *GBA*-associated Parkinsonism predisposes to an increased incidence of dementia (Fig. 1).

## Pathomechanisms in PD_GBA_

Experimental evidence from cell models suggests that *GBA* variants result in disrupted protein folding of GCase in the endoplasmic reticulum (ER), impaired trafficking of GCase from the ER to Golgi and ultimately in lower lysosomal GCase enzyme activity. This in turn causes a build-up of glucosylceramides (GlcCer) and glucosylsphingosines (GlcSph) (Beutler 1992) and impairs lysosomal function and thereby the degradation of α-synuclein (Mazzulli et al. 2011).

### *GBA* variants predispose to accelerated α-synuclein aggregation and Lewy-body pathology

Post-mortem studies show enhanced aggregation and propagation of α-synuclein not only in the substantia nigra and putamen but also wide-spread neocortical Lewy-body pathology in brain tissue of PD_GBA_ and DLB_GBA_ (Neumann et al. 2009; Gundner et al. 2019).

The field of PET imaging markers to assess the cerebral load of α-synuclein *in-vivo* is difficult. However, this month [03(2022)] first positive results were reported at the AD/PD Conference for a new PET tracer developed by AC Immune to distinguish multiple system atrophy (MSA) from healthy controls and patients with other forms of α-synuclein (PD, DLB). Therefore, research in PD has focused on CSF. Yet, it is unclear whether CSF profiles of α-synuclein species reflect brain pathology. Cross-sectional and longitudinal analyses in PD_GBA_wildtype_ and PD_GBA_ demonstrated decreased CSF levels of total α-synuclein compared to healthy controls with the highest decrease in PD_GBA_ patients carrying severe variants (Malek et al. 2014; Mollenhauer et al. 2019; Lerche et al. 2020, 2021a). Correspondingly, the same pattern was also reported in patients with DLB_GBA_ (Lerche et al. 2019a). However, a substantial inter-individual variability and overlap with healthy controls is seen, so that CSF levels of total α-synuclein are not ideal. Recently, the ultrasensitive assays real‐time quaking‐induced conversion (RT‐QuIC) and protein misfolding cyclic amplification (PMCA) have been successfully implemented. These assays exploit the seeding capacities of prion or prion-like proteins as an amplification strategy to reveal minute amounts of disease-specific protein aggregates in CSF (Fairfoul et al. 2016; Shahnawaz et al. 2017). Both methods are highly sensitive (88–96%) and specific (83–98%) for α-synuclein aggregates and Lewy-body pathology in PD and DLB as assessed in matched CSF/brain samples compared to healthy controls and other forms of dementia and parkinsonism (Rossi et al. 2020; Kang et al. 2019). However, histopathological findings in some genetic forms of PD are remarkably variable. While PD_GBA_ show extensive Lewy-body pathology, most PD patients with bi-allelic mutations in the recessive gene *PRKN* (PD_recessive_bi-allelic_) show nigral degeneration without Lewybodies (Schneider and Alcalay 2017). Also, histopathology in PD patients with *LRRK2* mutations (PD_LRRK2_) is variable, including typical Lewy-body pathology, misfolded tau deposition, or nigral degeneration without Lewy-body (Zimprich et al. 2004; Heckman et al. 2016; Kalia et al. 2015). This prompted us to evaluate CSF α-synuclein seeding capacities with RT-QuIC in two large cohorts of PD and DLB patients enriched for genetic forms. Remarkably, PD_GBA_ (93%) and DLB_GBA_ (100%), especially those carrying severe variants, showed the highest percentage of positive α-synuclein seeding and the most pronounced α-synuclein seeding kinetics. In contrast, PD_recessive_bi-allelic_ did not show CSF α-synuclein seeding at all, whereas those carrying heterozygous mutations in these recessive genes showed less α-synuclein seeding than PD_wildtype_ (91%) with a reduced positivity rate of 59%. Also, PD_LRRK2_ showed a reduced rate of α-synuclein seeding (78%) compared to PD_wildtype_ (Brockmann et al. 2021). The heterogeneity in α-synuclein seeding activity among the different genetic forms mirrors histopathological findings in these cases and highlight the value of α-synuclein seeding activity as an *in-vivo* marker of Lewy-body pathology.

The accelerated cognitive decline PD_GBA_ makes this subgroup of PD a good model to study CSF profiles that are associated with cognitive impairment. In general, limbic and/or cortical Lewy-body pathology is hypothesized to be the main substrate forcing driving cognitive decline in PD (Aarsland et al. 2005). In more recent years, it became clear that a considerable proportion of PD patients who developed dementia in their disease course show concomitant amyloid-beta and tau pathology at autopsy in addition to the typical Lewy-body pathology (Halliday et al. 2008; Compta et al. 2011). Correspondingly, reduced CSF levels of Amyloid-beta_1-42_ (Aβ_1-42_) and/or elevated CSF levels of total-Tau (t-Tau) and phospho-Tau (p-Tau) have been reported to be associated with cognitive impairment in PD (Brockmann et al. 2015a, 2017; Lerche et al. 2019b; Kang et al. 2016). However, this seems not to be the case in PD_GBA_ as CSF levels of Aβ_1-42_, t-Tau, and p-Tau are similar to those seen in healthy control individuals. In light of the CSF profiles of reduced total levels of α-synuclein and the prominent α-synuclein seeding activity, the pronounced cognitive decline in PD_GBA_ is driven by α-synuclein aggregation and cortical Lewy-body pathology.

Taken together, these histopathological and CSF characteristics of predominant and accelerated α-synuclein-driven Lewy-body pathology make PD_GBA_ and DLB_GBA_ a role model to study pathways leading to α-synuclein aggregation and highlight these patient cohorts as prime candidates for clinical trials targeting α-synuclein.

### GCase deficiency and α-synuclein aggregation

Heterozygous variants in the *GBA* gene are associated with a reduction of GCase protein levels and GCase enzyme activity in cell and animal models as well as in a variety of patient-derived biomaterials (Lerche et al. 2021a; Alcalay et al. 2015, 2020; Schondorf et al. 2014; Paciotti et al. 2019). Again, the degree of reduction is dependent from variant severity. Interestingly, GCase activity is also reduced in PD_GBA_wildtype_, albeit to a lesser degree (Parnetti et al. 2017).

There is reasonable evidence from different cell models including induced pluripotent stem (IPS) cell-derived human dopaminergic midbrain neurons and human midbrain organoids that deficiency of the GCase enzyme is paralleled by increased levels of intracellular α-synuclein, specifically α-synuclein species susceptible to aggregation such as high molecular weight and decreased tetramer/monomer ratio (Schondorf et al. 2014; Kim et al. 2018; Magalhaes et al. 2016; Aflaki et al. 2016; Mazzulli et al. 2016; Jo et al. 2021). Correspondingly, post-mortem studies in PD_GBA_ and DLB_GBA_ and to a lesser degree also in PD_GBA_wildtype_ and DLB_GBA_wildtype_ revealed that reduced GCase protein levels and reduced GCase enzyme activity are accompanied by increased levels of intracellular α-synuclein. Notably, these findings are not restricted to the substantia nigra and putamen but also identified in cortical regions (Murphy et al. 2014; Gegg et al. 2012; Moors et al. 2019; Gundner et al. 2019).

More specifically, it is suggested that lysosomal GCase and α-synuclein are linked in a bidirectional pathogenic loop as shown in cell cultures and IPS cell-derived dopaminergic midbrain neurons: (I) functional loss of GCase activity compromises lysosomal degradation of α-synuclein and promotes its aggregation. (II) α-Synuclein itself inhibits the activity of GCase (Mazzulli et al. 2011; Schondorf et al. 2014). Consequently, PD_GBA_ fulfill both conditions of this bidirectional loop in parallel leading to a self-reinforcing mechanism. Thereby, α-synuclein aggregation and propagation might be accelerated which possibly explains the wide-spread neocortical Lewy-body pathology and rapid clinical progression.

However, this bidirectional loop between GCase and α-synuclein might be oversimplified, since we have clear evidence for a more complex impairment of the autophagy–lysosomal pathway including disrupted macroautophagy with reduced fusion of autophagosomes with lysosomes and decreased expression/activity of other proteolytic lysosomal enzymes such as cathepsin B and D (Aflaki et al. 2020; Blauwendraat et al. 2020a; Lerche et al. 2021b).

### Disturbance in sphingolipid homeostasis and α-synuclein aggregation

Adding to the complexity of the underlying pathophysiology are additional alterations of intracellular and membrane-associated sphingolipid homeostasis. In GD patients, GD post-mortem brain studies, and IPS cell-derived human dopaminergic midbrain neurons with bi-allelic and heterozygous *GBA* variants, GCase deficiency results in accumulation of the GCase substrates GlcCer and GlcSph. PD_GBA_ patients, specifically PD_GBA_ with severe variants, show not only elevated levels of the GCase substrates GlcCer and GlcSph in CSF and plasma but also increased CSF levels of downstream-products (Cer) and by-products (SPA, S1P) when compared to healthy controls and PD_GBA_wildtype_ (Lerche et al. 2021a; Surface et al. 2022). Assessments in plasma from PD_GBA_wildtype_ as well as in aging mouse models further support findings that with decreasing GCase, activity levels of downstream/by-products are also elevated in addition to upstream substrates (Hallett et al. 2018; Mielke et al. 2013). In GD with pronounced GCase deficiency, GlcCer are alternatively processed to GlcSph and exit the lysosome into the cytosol (Hein et al. 2007; Elleder 2006; Ferraz et al. 2016). Cytosolic GlcSph is further hydrolyzed to ceramides, sphingosine, and sphingosine-1-phosphate. Recent studies highlight the role of ceramides and sphingosine-1-phosphate as key players in the regulation of cell death and survival with involvement in ER stress, autophagy, protein and lipid transport, exosome secretion with neurotoxic protein spreading, neuroinflammation, and mitochondrial dysfunction (Wang and Bieberich 2018). Data from *α-synuclein/GBA* transgenic mice and HEK cell cultures show that GlcCer, GlcSph, sphingosine, and sphingosine-1-phosphate promote the formation of oligomeric α-synuclein (Taguchi et al. 2017). Expanding these findings, recent data in human dopaminergic midbrain neurons suggest that conformational changes of α-synuclein towards an aggregation-prone pattern can be even induced by the presence of glycosphingolipids alone irrespective of GCase deficiency due to variants in the *GBA* gene (Zunke et al. 2018). Post-mortem studies show increased levels of GlcCer, GlcSph and ceramides in the substantia nigra and frontal cortex of PD_GBA_wildtype_ (Rocha et al. 2015a; Huebecker et al. 2019; Kurzawa-Akanbi et al. 2021). However, no differences were seen in the putamen of PD_GBA_ and PD_GBA_wildtype_ compared to controls (Gegg et al. 2012). More post-mortem studies with uniformly assessed brain regions and cell types as well as stratification according to *GBA* variant severity are needed to shed light on these seemingly discrepancies.

Enhanced activation of phosphocholine cytidyltransferase resulting in increased synthesis of phosphatidylcholine as major component of phospholipid cell membranes was reported in GD (Bodennec et al. 2002). Interestingly, alterations in the lipid bilayer composition of membranes cause impaired α-synuclein membrane binding and enhance aggregation-prone fibril formation (Piccinini et al. 2010). Combined ^1^H and ^31^P magnetic resonance spectroscopic imaging revealed that PD_GBA_ patients display a disturbed membrane phospholipid metabolism in the putamen and midbrain with reduced levels of the precursor choline and increased levels of the membrane-related phospholipid degradation product glycerophosphoethanolamine. These changes were accompanied by neuronal loss in these brain regions as measured by reduced levels of the neuronal marker *N*-acetyl-aspartate (Brockmann et al. 2012).

## Therapeutic targets in *GBA*-associated PD

Based on the knowledge of the molecular mechanisms underlying PD_GBA_, pathway-specific treatment options are beginning to emerge.

### GCase

The significant reduction of GCase protein levels and GCase enzyme activity offer a plausible therapeutic rational to either increase GCase protein levels or enhance enzyme activity. Unfortunately, intravenous enzyme replacement therapy is not possible due to insufficient central nervous system penetration.

Gene therapy with adeno-associated virus (AAV)-based vectors promoting *GBA* overexpression. This approach reduced α-synuclein accumulation, improved lysosomal function and lipid turnover, and attenuated deficits in working memory and fine motor performance in α-synuclein mutant/overexpressing wild-type and GD rodent models (Rocha et al. 2015b; Glajch et al. 2021; Sardi et al. 2011). The AAV9-based vector PR001 increased GCase activity, reduced glycolipid substrate accumulation, and improved motor deficits in two mouse models of GCase deficiency (Abeliovich et al. 2021). Based on these results, a phase 1/2a non-randomized clinical trial with a single administration of PR001 into the cisterna magna is currently under investigation in PD patients with at least one pathogenic *GBA* variant. The study duration is 5 years. During the first year, patients will be evaluated for safety, tolerability, immunogenicity, biomarkers, and clinical efficacy measures. Patients will continue to be followed for an additional 4 years to monitor safety and selected biomarker and efficacy measures (NCT04127578).

GCase-enhancing small-molecule chaperones refold misfolded GCase in the ER and promote proper trafficking, thereby increasing lysosomal GCase protein levels. Interestingly, experimental data in cell and animal models with *GBA* variants suggest that the expectorant Ambroxol increases GCase availability via such mechanism (Kopytova et al. 2021; Ambrosi et al. 2015; Maegawa et al. 2009; Magalhaes et al. 2018; McNeill et al. 2014; Yang et al. 2022; Migdalska-Richards et al. 2016). These findings led to a proof-of-principle phase 2 open-label study with Ambroxol in 17 PD patients with and without *GBA* variants. Ambroxol was well tolerated and CSF GCase protein levels as well as CSF levels of α-synuclein increased by 35% and 13%, respectively. However, CSF GCase enzyme activity decreased by 19% which might be explained by an inhibitory effect of Ambroxol on GCase activity within acellular human CSF with a neutral pH (Mullin et al. 2020).

More strikingly, a recent publication could show that the small-molecule S-181 increases wild-type GCase activity in iPSC-derived dopaminergic neurons not only from PD_GBA_ but also from PD_wildtype_ as well as from patients with other PD-related gene mutations in *LRRK2*, *DJ-1*, and *PARKN* who also had decreased levels of GCase activity. S-181 treatment of these PD iPSC-derived dopaminergic neurons partially restored lysosomal function and lowered accumulation of oxidized dopamine, GlcCer, and α-synuclein (Burbulla et al. 2019). These recent findings highlight not only the importance of lysosomal dysfunction in the pathophysiology of the prototype PD_GBA_ but also the significance of this pathway, possibly in concert with additional pathways such as mitochondrial dysfunction, for PD in general.

### Substrate reduction therapy

Substrate reduction therapy to reduce GlcCer production with penetration into the central nervous system is available for oral application in GD. Venglustat has been evaluated in a phase 2 randomized trial (MOVES-PD, NCT02906020) in PD_GBA_. The compound clearly reduced CSF levels of GlcCer in a dose-dependent manner in plasma and CSF. However, the study was stopped prematurely, since patients in the verum group showed enhanced clinical deterioration suggesting an off-target effect with possible anti-dopaminergic activity.

### Alpha-synuclein-targeting compounds

Targeting alpha-synuclein also seems a reasonable treatment option given the predominant α-synuclein aggregation and wide-spread Lewy-body pathology in PD_GBA_.

## Conclusion and outlook

*GBA*-associated PD is remarkable for several reasons. The phenotypical trajectories show a faster disease progression with pronounced early cognitive decline and a clear dependency based on mutation severity. Importantly, the development of dementia is not associated with Amyloid-β pathology as shown instead in a relevant proportion of PD without *GBA* variants but rather due to predominant α-synuclein aggregation. The identified pathophysiological mechanisms highlight GCase deficiency and lysosomal dysfunction resulting in disrupted glycosphingolipid homeostasis and ultimately impaired α-synuclein degradation with enhanced aggregation. Again, these are dependent on mutation severity and offer different targets for individualized treatment options. However, the failure of the MOVES-PD trial (NCT02906020) demonstrates the challenges we are facing in translational research. Findings from GD as typical and clearly defined young-onset lysosomal storage lipid disorder due to bi-allelic mutations in *GBA* are not simply transferable into PD, a multifactorial disease of the elderly with possibly additional contributing factors (e.g., mitochondrial dysfunction and lifetime environmental exposure). Specifically, the pathophysiological mechanisms of impaired glycosphingolipid homeostasis leading to impaired α-synuclein degradation need more investigation. In this context, longitudinal patient cohorts with repeated collections of biomaterials, ideally starting in the prodromal stage followed up until death with brain donation, might inform us on biomarkers that reflect the underlying pathological processes and possible read-outs for target engagement.

Future clinical trials in PD_GBA_ might incorporate the knowledge learned over the last years: (i) Patients should be stratified according to *GBA* variant severity with those carrying severe mutations to be preferentially included in proof-of-concept trials. (ii) The early cognitive decline based on predominant α-synuclein-driven pathology offers the opportunity to address PD-associated dementia with disease-modifying agents in a clearly defined prodromal phase preceding dementia and based on clear biological stratification.
